# Cancroid Stomatitis

**Published:** 1880-02

**Authors:** A. F. M’Lain


THE DENTAL REGISTER.

VOL. XXXIV.] FEBRUARY, 1880. [No. 2.

### CANCROID STOMATITIS.

BY A. F. M'LAIN, M.D., D.D.S.

MR. PRESIDENT AND GENTLEMEN:—I desire to call your attention on this occasion to a form of sore mouth, which is so common as scarcely ever to elicit any notice from the profession, but which, at times, causes considerable pain and distress. I refer to what is usually known as "Aphthæ," to which, however, the generic term of "*Cancroid Stomatitis*" is more applicable, as it avoids the confusion of ideas which so frequently occurs when the word aphthæ is used. Being derived from two Greek verbs, both having the same orthography, (apto)—one meaning "*to set on fire*," the other to "*bind to*" or "*fasten upon*," has given rise among authors to many contradictory descriptions under a common name, as one or another species of this class of disease is treated.

Now, all of these several species of aphthæ, commonly known as *thrush* or *mugnet*, *follicular inflammation*, *cancrum oris* and *gangrena oris*, although they may individually present some slight diversity in general features, yet they all bear a family type, and are essentially of a cancroid nature. The difference, which exists between them, consists more, however, in their extent, degree or locality, and in the circumstances with which they are attended.

Viewed as a type, the various species of aphthæ present a characteristic, cancrous, degenerate sore or ulcer, varying from the smallest point to a confluent mass, covering a large extent of surface, which surface has a pasty or exudative appearance. The color varies in the different kinds of aphthæ from a misty velvety white of hoar frost to the dirty yellow of scrofulous pus. Their habitat, according to circumstances, is to be found upon the mucous membrane of the mouth, lips, cheeks, fauces and æsophagus ; and sometimes, particularly in the case of thrush, it may invade various portions of the alimentary or respiratory tract.

Thrush, as you well know, is a disease more peculiar to the time of lactation in infancy, while the other forms belong more especially to the dentitional period. This disease begins with an erythematic inflammation, on which, however, soon appear white spots, which, if not arrested, spread, assuming those curdy, velvety white patches already mentioned.

*Follicular inflammation* has its origin, as its name indicates, in an inflammation of the crypts and follicles of the mucous membrane of the mouth and tongue, which may terminate by resolution, but which, more frequently, under favorable conditions, degenerates into ulcerated points, which, in a short time coalesce and form, according to the severity of the case, surfaces from a curdy white to a dirty brown color.

*Cancrum oris* and *gangrena oris* present nearly the same features, excepting that the former is a phagedenic or spreading ulcer, the latter a sloughing one ; both being more localized in their inception than the others.

These several varieties of canker, for it must be apparent that aphthæ is nothing more nor less than a canker, having their causes from bad or insufficient nourishment, impure air and uncleanliness, or all combined, by which means, perhaps a more marked expression is given to the severer forms. It is therefore evident that aphthæ, in all its varieties, is due to a depraved condition of the system.

The kind of stomatitis to which your attention is now especially directed, is likewise a canker, but is a disease more peculiar to adults, and bears a close analogy to those which appear in younger subjects. Like them, it may occur on the gums, tongue and internal parts of the lips or cheeks. Like thrush, it may begin with small, white points, which in time coalesce. Or, as follicular inflammation commences in the crypts and follicles of the tongue or other portions of the mucous membrane of the mouth, and gradually form exudate patches ; or, in the manner of cancrum oris, become from a single point, a spreading or phagedenic ulcer, with red inverted edges and a characteristic elevated or depressed white center. As a general rule, the disease we refer to involves no serious consequences, yielding readily to the appropriate remedies, but occasionally it is very persistent, and is thus capable of proving a great source of annoyance, both to patient and the adviser.

I look upon the disease as being the same, and arising from the same causes, as those belonging to infancy; but as it occurs in subjects of stronger physical powers and dependent less upon cachexia, it loses its force and does not produce similar constitutional phenomena, or assume forms as aggravated as those of early life. But there is no doubt that the disease results from an ill condition of the *primæ-viæ* or from a cachectic disposition of the system.

These little canker sores will very frequently develop themselves suddenly and in a few days gradually subside of themselves, withont the intervention of remedial measures. But sometimes they evince not only a considerable degree of tenacity, but they exhibit a great phagedenic tendency. Generally, in these cases, one or two applications of diluted aromatic sulphuric acid or simply diluted sulphur or nitric acid will suffice to eradicate those aphthous sores thoroughly. At other times, however, both local and general treatment will be required.

A case in point will serve to illustrate the persistency which the disease sometimes acquires, also how its attending symptoms vary or otherwise take on complicated forms.

Some years ago, a gentleman aged about twenty-eight years, of a sanguino-billious temperament, consulted me for an aphthous ulceration in the vicinity of an inferior wisdom tooth, which was short and partially covered by the gum. Cauterization and other remedial means having failed to procure relief, and the parts immediately connected with the tooth becoming implicated, extraction of the organ was resorted to with a decided amelioration in the symptoms, but this was soon followed by the development of aphthous spots on remote and previously healthy tissues, both anteriorily and posteriorily. Under the use of sulphuric acid, applications once a day and a wash, the base of which was tannic acid, three or four times daily, the sores had almost if not quite cicatrized, and the parts assumed a healthy aspect. Just about this time business called the gentleman to the country, and after three or four weeks absence he returned, complaining greatly. An examination disclosed a large ulceration behind the superior wisdom tooth of the same side, several aphthous patches on the velum and posterior pillars, and also on the buccal surface of the cheek, opposite the first molar tooth. Immediate resort was had to the acid applications, every morning, followed by annointing the parts with a weak preparation of Lugal's solution of iodine, combined with tannic acid and glycerine, he, in the meantime, using through the day a wash containing permanganate of potash and chlorinated soda, with a few drops of creosote. This treatment would so far control the disease that, at his afternoon visit, a marked improvement would be manifest, but the next morning it would be found that a relapse to the former condition had taken place. Thus for a number of days, we had constantly intermitting relapses and partial recoveries. When, supposing that a syphilitic taint might have something to do with the case, he was put upon a course of iodide of potassium, in addition to the local treatment, which resulted in a short time in a perfect cure.

At about the same time, the following year, this gentleman came to me with two cancroid sores, one over the left superior central and the other a little behind the latteral incisor teeth. Recourse was again had to the same treatment which had proven so successful before, but without the least benefit. On the contrary, the cancroid patches had coalesced and assumed a phagedenic form, extending in every direction. Various combinations of washes were tried without effect. Emolient drinks and mild salines did some good, but all caustics actually aggravated the disease—nitrate of silver more so than the sulphate of copper. After exhausting the pharmacopæia almost for remedies with little effect, and seeing the patient was getting apprehensive and nervous, he was put upon the use of quinine and iron, and the sores dressed twice a day with simply pulverized dried alum, and to our surprise and satisfaction, in less than a week the parts were so completely restored to health that on the closest inspection not the slightest trace of disease could be detected.

—*Trans. Cal. State Dental Asso.*

